# Supplementary material for: Reconstruction Set Test (RESET): A computationally efficient method for single sample gene set testing based on randomized reduced rank reconstruction error
Source: PLoS Comput Biol. 2024 Apr 29;20(4):e1012084. doi: 10.1371/journal.pcbi.1012084 (PMC11081506; doi:10.1371/journal.pcbi.1012084)
Supplement: S1 Text — (PDF) [file pcbi.1012084.s001.pdf]

Reconstruction Set Test (RESET): a computationally efficient method for single sample gene set testing based on randomized reduced rank reconstruction error
Supplemental Information

H. Robert Frost *

Contents

1 Supplemental Methods **3**
- 1.1 Randomized numerical linear algebra methods . . . 3
- 1.2 RESET-Seurat integration . . . 3
- 1.3 Simulation study design . . . 4
- 1.4 Real data analysis design . . . 7

2 Supplemental Results **8**
- 2.1 Simulated results for overlapping gene sets . . . 8
- 2.2 Computational performance on simulated data . . . 11
- 2.3 Human PBMC analysis . . . 12
- 2.4 Mouse brain cell analysis . . . 16
- 2.5 Human cord blood cell analysis . . . 18

List of Tables

- A Top 20 BioCarta pathways according to overall RESET score for the PBMC data set with per-variable adjustment. . . . 12
- B Top 20 BioCarta pathways according to overall RESET score for the human cord blood data set using Seurat log-normalization. . . . 18
- C Top 20 BioCarta pathways according to overall RESET score for the human cord blood data set using Seurat log-normalization with per-variable adjustment. . . . 18
- D Top 20 BioCarta pathways according to overall RESET score for the human cord blood data set using SCTransform normalization. . . . 19
- E Top 20 BioCarta pathways according to overall RESET score for the human cord blood data set using SCTransform normalization with per-variable adjustment. . . . 19

*rob.frost@dartmouth.edu, Department of Biomedical Data Science, Geisel School of Medicine, Dartmouth College, Hanover, NH 03755

List of Algorithms

A Randomized column space basis generator (randomColumnSpace) 3
B Randomized SVD (randomSVD) 4

List of Figures

A Classification performance of RESET.det, RESET.ran, VAM, GSVA, ssGSEA, and PLAGE on scRNA-seq data simulated according to the block design for overlapping and unequally sized gene sets as detailed in SI Section 1.3. Each panel illustrates the relationship between the area under the receiver operating characteristic curve (AUC) and one of the simulation parameters. The vertical dotted lines mark the default parameter value used in the other panels. Error bars represent the standard error of the mean. 8
B Overall classification performance of RESET.det, RESET.ran and RESET.pervar on scRNA-seq data simulated according to the block design for overlapping and unequally sized gene sets as detailed in SI Section 1.3. Each panel illustrates the relationship between the area under the receiver operating characteristic curve (AUC) and one of the simulation parameters. The vertical dotted lines mark the default parameter value used in the other panels. Error bars represent the standard error of the mean. 9
C Classification performance of RESET.det, RESET.ran, RESET.pervar, VAM, GSVA, ssGSEA, and PLAGE on scRNA-seq data simulated according to the pure competitive design for overlapping and unequally sized gene sets as detailed in SI Section 1.3. Each panel illustrates the relationship between the area under the receiver operating characteristic curve (AUC) and one of the simulation parameters. The vertical dotted lines mark the default parameter value used in the other panels. Error bars represent the standard error of the mean. 10
D Average execution time of RESET.det, VAM, GSVA, ssGSEA, and PLAGE relative to RESET.ran. Relative values are plotted on the $\log_{10}$ scale. Execution times were computed on data simulated according to the procedure outlined in Section 1.3 for the block design. Error bars represent the standard error of the mean. 11
E Projection of PBMC scRNA-seq data onto the first two UMAP dimensions. Each point in the plot represents one cell. Cluster annotations reflect estimated cell type. 13
F Projection of RESET BioCarta scores for the PBMC scRNA-seq data onto the first two UMAP dimensions where UMAP was executed on the top 30 PCs of the RESET score matrix. Each point in the plot represents one cell.Annotations reflect estimated cell type according to the scRNA-seq clusters. 14
G Heatmap visualization of the RESET cell-specific scores for the top five BioCarta pathways most enriched in each cluster of the PBMC scRNA-seq data according to the log2 fold-change in the mean RESET score of cells in the cluster relative to cells not in the cluster. Note that gene sets only appear once in the heatmap even if they are among the top five sets for multiple clusters. 15
H Projection of mouse brain scRNA-seq data onto the first two UMAP dimensions. Each point in the plot represents one cell, which are colored and labeled accounting to the output from unsupervised clustering. 16

I Visualization of cluster-level Gene Ontology biological process term enrichment as computed using either VAM or RESET scores for the mouse brain scRNA-seq data. 17
J Visualization of cell type BioCarta pathway enrichment for select immune cell types as computed using either VAM or RESET scores on the human cord blood scRNA-seq data using Seurat log-normalization. 20
K Visualization of cell type BioCarta pathway enrichment for select immune cell types as computed using either VAM or RESET scores on the human cord blood scRNA-seq data using SCTransform normalization. 21

1 Supplemental Methods

1.1 Randomized numerical linear algebra methods

The randomized RESET algorithm depends on two underlying RNLA functions: a randomized technique for computing an orthonormal basis for the column space of a matrix and, building on that method, a randomized SVD algorithm. These two RNLA techniques, defined in Algorithms S1 and S2 below, follow the general structure of the randomized rangefinder and randomized SVD algorithms in Martinsson et al. [1]. For clarity, we are not including power iteration functionality in Algorithms S1 or S2 (see Martinsson et al [1] or Erichson et al. [2] for details on power iteration functionality). We are also showing just the use of $N(0,1)$ random variables for creation of the sketch matrix. While the RESET method supports both $N(0,1)$ and $U(0,1)$ RVs, empirical studies have found that performance of randomized methods is generally insensitive to the choice of statistical distribution [1]. Readers interested in the theoretical and computation properties of these methods or the broader foundations/applications of RNLA are encouraged to read the excellent survey by Martinsson et al. The paper by Erichson et al [2] associated with the *rsvd* R package provides a shorter introduction to these methods with a specific focus on their programmatic implementation and performance benefits relative to truncated algorithms.

Algorithm A Randomized column space basis generator (randomColumnSpace)

Inputs:

- $\mathbf{X}$: $n \times p$ matrix
- k: Rank for computed column space basis
- d: Additional dimensions to compute

Output:

- $\mathbf{Q}$: $n \times k$ orthonormal matrix whose k columns for a basis for the column space of $\mathbf{X}$.

1: $\mathbf{O} \in \mathbb{R}^{p \times (k+d)}, \mathbf{O}[i,j] = \mathcal{N}(0,1)$ ▷ Create an $p \times (k+d)$ matrix of independent standard normal random variables

2: $\mathbf{Y} = \mathbf{XO}$ ▷ Compute an $n \times (k+d)$ sketch matrix Y whose columns are approximately independent linear functions of the columns of $\mathbf{X}$

3: $\mathbf{Q} = qr(\mathbf{Y})[, 1:k]$ ▷ Compute a $n \times k$ orthonormal basis for the columns space of $\mathbf{X}$ via a column-pivoted QR decomposition of $\mathbf{Y}$

return Q

1.2 RESET-Seurat integration

To support the analysis of scRNA-seq data, the RESET R package includes a wrapper function (*resetForSeurat()*) that enables direct integration with the popular Seurat framework [3]. This

Algorithm B Randomized SVD (randomSVD)

Inputs:

- $\mathbf{X}$: $n \times p$ matrix
- k: Target rank for the computed SVD
- d: Additional dimensions to compute

Outputs:

- $\mathbf{U}$: $n \times k$ orthonormal matrix of left singular vectors
- $\mathbf{\Sigma}$: $k \times k$ diagonal matrix of singular values
- $\mathbf{V}$: $p \times k$ orthonormal matrix of right singular vectors

1: $\mathbf{Q} = \text{randomColumnSpace}(\mathbf{X}, k, d)$ ▷ Use randomColumnSpace function defined in Algorithm S1 to compute a rank k orthonormal basis for the column space of $\mathbf{X}$
2: $\mathbf{B} = \mathbf{Q}^T\mathbf{X}$ ▷ Project $\mathbf{X}$ onto $\mathbf{Q}$
3: $\mathbf{B} = \mathbf{U}_B\mathbf{\Sigma}\mathbf{V}^T$ ▷ Compute the non-randomized SVD of $\mathbf{B}$
4: $\mathbf{U} = \mathbf{Q}\mathbf{U}_B$ ▷ Compute left singular vectors of $\mathbf{X}$
return $\mathbf{U}$,$\mathbf{\Sigma}$,$\mathbf{V}$

integration supports both log-normalization and SCTransform [4] normalization and assumes that PCA has already be performed via the Seurat *RunPCA()* function. To use an alternative data subset or transformation for measuring reconstruction error, the underlying *reset()* function must be called directly. The $\mathbf{S}$ matrix of cell-level gene set scores output by RESET is saved as a new Seurat assay named "RESET", which enables the visualization and further analysis of these scores using Seurat framework, e.g., the *FeaturePlot()* and *FindMarkers()* functions. The vector $\mathbf{v}$ of overall gene set scores is saved in the feature metadata column named "RESET". See the RESET R package documentation and vignettes for further details and examples.

1.3 Simulation study design

To evaluate the performance of the RESET method, we applied it to simulated sparse count data with statistical charateristics similar to those found in scRNA-seq data. The R code that implements this simulation design and generates the various result figures is included along with a brief README file on the paper website: https://hrfrost.host.dartmouth.edu/RESET/RESET_simulation_logic.zip. In particular, we simulated $\mathbf{X}$ matrices representing normalized scRNA-seq data for 2,000 cells and 500 genes as follows:

1. **Simulate non-informative counts**: Populate all entries of $\mathbf{X}$ with independent negative binomial random variables with a random mean and overdispersion of 100. The overdispersion value of 100 is based on the finding by Lause et al. [5] that scRNA-seq counts can be effectively modeled by a negative bionomial distribution with flexible mean and fixed overdispersion of 100. The random negative binomial mean is generated as $\mu = 0.01 + \alpha$ where $\alpha \sim Exp(\lambda = 4.5)$. The constant of 0.01 is included to provide independent control over the maximum expected gene sparsity (this both mimics quality control filtering and allows the impact of sparsity on performance to be more accurately assessed). This process generates a range of mean values with an expectation of 0.23 and expected fold-change between the minimum and maximum means for 500 genes of 145. The expected mean of 0.23 is similar to the mean value for unnormalized counts in the PBMC3k scRNA-seq data used for the real data analysis (see Section 1.4 for details).
2. **Simulate counts for informative genes**: Update the columns in $\mathbf{X}$ corresponding to infor-

mative genes to be correlated with a larger mean. While the overdispersion parameter was kept at 100, the mean for these entries was generated as $\mu_{info} = \mu_c + \alpha_{info}$ where μ_c varies between 0 and 0.4 with a default of 0.2 and $\alpha_{info} \sim Exp(\lambda_{info})$ with λ_{info} varying between 0 and 8.5 with a default of 4.5. In the simulation result plots, the variation in μ_c is captured by the "Mean" plot and the variation in λ_{info} is captured in the "Rate" plot. This generates informative gene counts with a larger default expected mean (0.42) and a much larger variation in maximum sparsity and expected fold-change between minimum and maximum values, which is consistent with the larger mean value of genes considered informative in real scRNA-seq data, i.e., genes with a large proportion of biological variance.

3. **Simulate correlated/inflated counts for informative genes/cells**: A subset of the informative gene counts that correspond to informative cells are then updated to be correlated and have inflated mean values. This yields a pattern of differential correlation/expression between informative and non-informative cells for these genes. The inter-gene correlation for informative genes/cells was varied between 0 (the no differential correlation case) and 0.9 (this was accomplished using a Gaussian copula prior to generating marginal negative binomial values). The informative means generated as $\mu_{info} = \mu_c + \alpha_{info}$ were inflated for these counts by an inflation factor β, i.e., $\mu_{inflated} = \beta * \mu_{info}$ that varied between 1 and 2.2 with a mean of 1.6.
4. **Normalize**: Perform log-normalization of the count data to mirror Seurat's log-normalization procedure, i.e., divide the counts by the total for each cell, multiply by a scale factor of 500, add a pseudocount of 1 and take the natural log. The scale factor of 500 for 500 genes was selected to generate similar values as the scale factor of 10,000 used by Seurat on datasets with approximately 20,000 genes.

The number of informative genes/cells followed one of three structures:

1. **Block**: For this scenario, the informative genes/cells correspond to a strict subset of the genes/cells. The number of informative genes matched the size of the first gene set, which varied between 10 and 90, and the number of informative cells varied between 25 and 425. Because the informative gene/cell block does not include all of the genes or all of the cells, it generates a pattern that can be detected by both self-contained and competitive methods. Results for this scenario are shown in main manuscript Figures 1 and 4 and in SI Figures S1 and S2.

2. **Complex**: This scenario captures a more complex internal gene set structure with two correlated blocks of genes with distinct mean expression patterns. Specifically, the genes in the first set are split into two groups that contain 75% and 25% of the set genes respectively. The first 75% of the set genes are simulated using a structure identical to that used for the block design as described above, i.e., mean inflation and correlation hold just for the informative cells. The last 25% of the set genes are simulated with no differential expression, i.e, the expected value of the simulated counts is identical for these across all cells and matches that for non-informative cells, and an elevated correlation structure that holds for all cells, i.e., the genes have elevated inter-gene correlation within the 25% block and this elevated correlation holds across all cells.

3. **Pure self-contained**: For the pure self-contained case, all 500 genes are considered informative. This generates a pattern that can be detected by self-contained methods but not by competitive methods. Results for this scenario are shown in main manuscript Figure 2.

4. **Pure competitive**: For the pure competitive case, all 2,000 cells are considered informative. This generates a pattern that can be detected by competitive methods but not by self-contained methods. Results for this scenario are shown in main manuscript Figure 3 and in SI Figure S3.

A total of 50 $\mathbf{X}$ matrices were simulated for each unique combination of simulation parameters for each of the scenarios. These simulated $\mathbf{X}$ matrices were used to evaluate the classification and computational performance RESET and the comparison methods detailed in the main manuscript. For this evaluation, three different gene set collection structures were used:

1. **Single gene set**: Just a single gene set is used that always includes a subset of informative samples, i.e., samples with a mixture of differential expression and correlation. Results for a single gene set and the block design are shown in main manuscript Figure 1 and for a single gene set and for the pure self-contained design in main manuscript Figure 2.

2. **Disjoint and equally sized collection**: For this scenario, five equally sized and disjoint gene sets were evaluated. As detailed above, set size was varied between 10 and 90. Only the first of the five sets includes informative samples, which enables the AUC to be computed. Results for disjoint and equally sized gene sets and the block design are shown in main manuscript Figures 3 and 4.

3. **Overlapping and unequally sized collection**: For this scenario, five overlapping gene sets with different sizes were evaluated. If the target set size is s, these gene sets are created as follows:

   - Set 1: Contains the first s genes, i.e., all of the informative/perturbed genes.
   - Set 2: Contains genes with indices from $s+1$ to $2s$, i.e., same size as Set 1 but does not include any informative genes.
   - Set 3: Contains the first $s/2$ genes and genes with indices $s+1$ to $s+s/2$, i.e., same size as Set 1 but only half of the genes are informative.
   - Set 4: Contains the first $2s$ genes, i.e., twice the size of Set 1 with all the informative genes and an equal number of uninformative genes.
   - Set 5: Contains first $3s$ genes, i.e., three times the size of Set 1 with all the informative genes and twice the number of uninformative genes.

   For the computation of AUC, it is assumed that Set 1 should have the largest score. Results for overlapping and unequally sized gene sets and the block design are shown in SI Figures S1 and S2 and for the pure competitive design in SI Figure S3.

Three different versions of RESET, RESET.det, RESET.ran and RESET.pervar, were evaluated based on the setting of the *random.threshold* and *per.variable* parameters. For RESET.det, which stands for deterministic RESET, *random.threshold* was set to 90, which forces the use of column-pivoted QR decomposition, and *per.var* was set to false. RESET.pervar used the same *random.threshold* value but set *per.var* to true to adjust the scores according to gene set size. For RESET.ran, which stands for randomized RESET, *random.threshold* was set to 9, which forces the use of the randomized column space basis generator, and *per.var* was set to false. All three RESET variants were realized by calling the *resetViaPCA()* function in version 0.2.1 of the RESET R package with the following settings for all parameters except *random.threshold* and *per.var*: *num.pcs=10, pca.buff=10, pca.q=2, k=10, k.buff=0, q=0, test.dist="normal", norm.type="2"*. Single sample

gene set scores were computed using each evaluated technique for all simulated **X** matrices and AUC values were calculated based on the ability of each method to give modified cells a higher score than unmodified cells. Overall classification performance was also assessed for the two RESET versions (other methods could not be evaluted since they do not generate overall scores). To assess overall classification performance, a total of five equally sized sets were scored with only the first set containing modified counts. The same five set design was also used to assess classification performance for the pure competitive scenario, i.e., single sample scores for all five sets were included in the AUC computation. Note that execution time for RESET did not include generation of the PC projections via randomized SVD since that is only called once for all evaluated gene sets so would disporportionately impact the results when only a single gene set is evaluated. The time required for computing the PC projections is also not a factor for Seurat-based analyses since PCA is typically performed regardless for clustering and visualization.

1.4 Real data analysis design

To evaluate RESET on real transcriptomic data, we analyzed three public scRNA-seq data sets available from 10x Genomics: the 2.7k human PBMC data set used in the Seurat Guided Clustering Tutorial [6], an 11.8k mouse brain cell data set generated on the combined cortex, hippocampus and sub ventricular zone of an E18 mouse [7], and an 8.6k human cord blood data set. These data sets were selected in part because two of them also used in the VAM paper so provide a direct measure of comparative performance. Similar to the rationale listed in the VAM paper, these data sets are representative of small and medium sized experiments and capture gene expression profiles for three different complex cells populations (peripheral blood, cord blood and neural cells) from two organisms (human and mouse) that comprise a large percentage of existing scRNA-seq data. Preprocessing, quality control (QC), normalization and clustering of the PBMC data set matched the Seurat Guided Clustering Tutorial. Specifically, the Seurat log-normalization method is used followed by application of the *vst* method for decomposing technical and biological variance. Preprocessing and QC of the PBMC data yielded an **X** matrix of normalized counts for 14,497 genes and 2,638 cells.

Processing of the mouse brain data followed similar quality control metrics (at least 200 features per cell, non-zero values in at least 10 cells for genes, proportion of mitochondrial reads less than 10% [8]) with Uniform Manifold Approximation and Projection (UMAP) [9] used for dimensionality reduction and clustering performed with Seurat's implementation of shared nearest neighbor (SNN) modularity optimization [10]. Normalization of the mouse brain data was performed using SCTransform [4] rather than log-normalization to assess RESET performance for both of the supported Seurat normalization approaches. Preprocessing and QC of the mouse brain data yielded an **X** matrix of normalized counts for 32,850 genes and 9,320 cells.

Processing of the cord blood also followed similar quality control metrics (at least 200 features per cell, non-zero values in at least 10 cells for genes, proportion of mitochondrial reads less than 5% [8]). Cell type annotations were based on the CITE-seq abundance for surface proteins as provided by the *cbmc* SeuratData data set. Normalization of the cord blood data was performed using both log-normalization and SCTransform [4] to explore the impact of normalization method on RESET. Preprocessing and QC of the cord blood data yielded an **X** matrix of normalized counts for 20,501 genes and 7,723 cells.

For these analyses, the gene set matrix **A** was populated using the human C2.CP.BIOCARTA (BioCarta, 292 gene sets), and the mouse C5.BP (Gene Ontology Biological Processes, 7,751 gene sets) collections from v2023.1 of the Molecular Signatures Database (MSigDB) [11]. These MSigDB collections represent two widely used groups of curated gene sets: BioCarta [11], and the biological

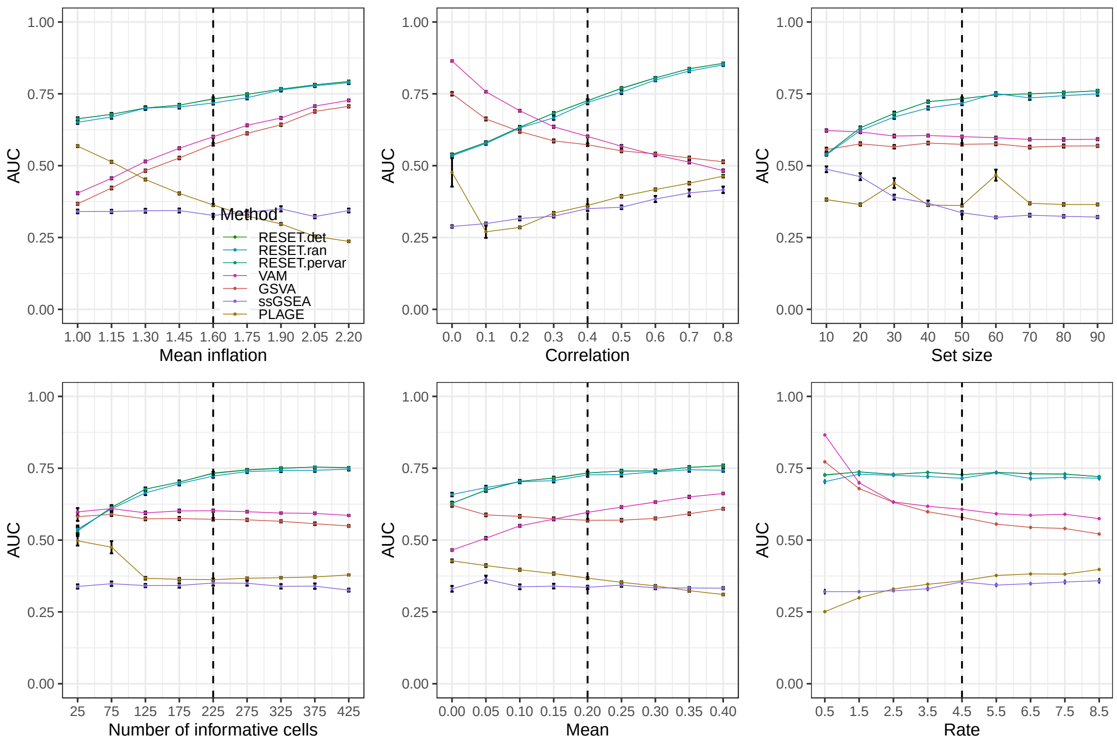

Figure A: Classification performance of RESET.det, RESET.ran, VAM, GSVA, ssGSEA, and PLAGE on scRNA-seq data simulated according to the block design for overlapping and unequally sized gene sets as detailed in SI Section 1.3. Each panel illustrates the relationship between the area under the receiver operating characteristic curve (AUC) and one of the simulation parameters. The vertical dotted lines mark the default parameter value used in the other panels. Error bars represent the standard error of the mean.

process branch of the Gene Ontology [12]. Prior to performing gene set testing, the Entrez gene IDs used by MSigDB were converted to Ensembl IDs using logic in the Bioconductor *org.Hs.eg.db* and *org.Mm.eg.db* R packages. The **X** and **A** matrices were then filtered to only contain genes present in both matrices (13,714 genes for the PBMC data and 16,425 genes for the mouse brain data). Finally, the **A** matrix was filtered to remove sets with fewer than 5 or more than 200 members. Enrichment of gene sets for specific scRNA-seq clusters was performed using a Wilcoxon rank sum test as implemented by the Seurat *FindMarkers()* method.

Execution of RESET for all of these data sets was performed using the *resetForSeurat* method in v0.2.1 of the RESET R package with the following parameter settings: *num.pcs=15, k=5, k.buff=0, q=0, random.threshold=30, test.dist="normal", norm.type="2"*.

2 Supplemental Results

2.1 Simulated results for overlapping gene sets

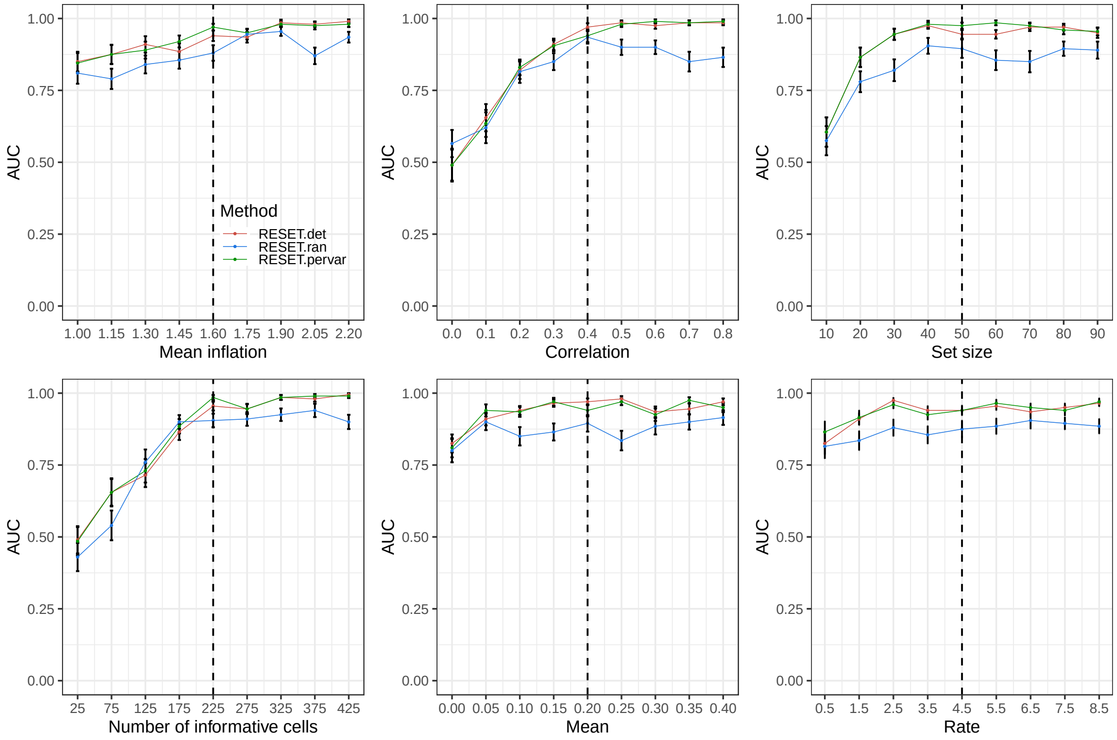

Figure B: Overall classification performance of RESET.det, RESET.ran and RESET.pervar on scRNA-seq data simulated according to the block design for overlapping and unequally sized gene sets as detailed in SI Section 1.3. Each panel illustrates the relationship between the area under the receiver operating characteristic curve (AUC) and one of the simulation parameters. The vertical dotted lines mark the default parameter value used in the other panels. Error bars represent the standard error of the mean.

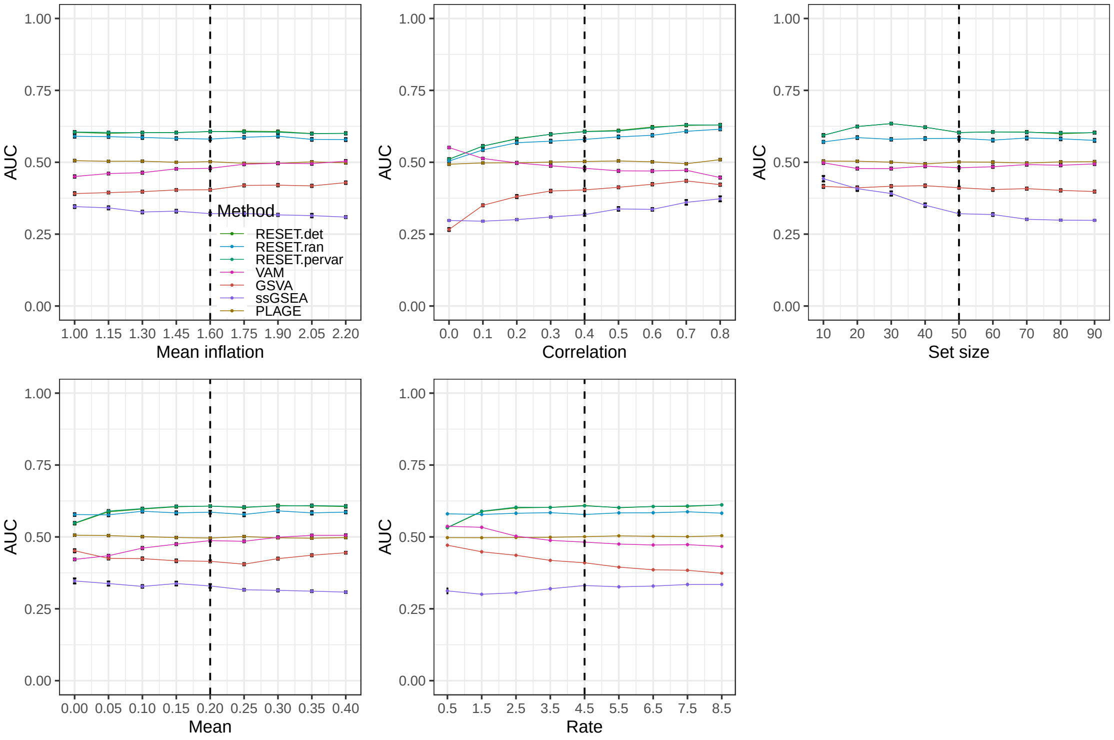

Figure C: Classification performance of RESET.det, RESET.ran, RESET.pervar, VAM, GSVA, ssGSEA, and PLAGE on scRNA-seq data simulated according to the pure competitive design for overlapping and unequally sized gene sets as detailed in SI Section 1.3. Each panel illustrates the relationship between the area under the receiver operating characteristic curve (AUC) and one of the simulation parameters. The vertical dotted lines mark the default parameter value used in the other panels. Error bars represent the standard error of the mean.

2.2 Computational performance on simulated data

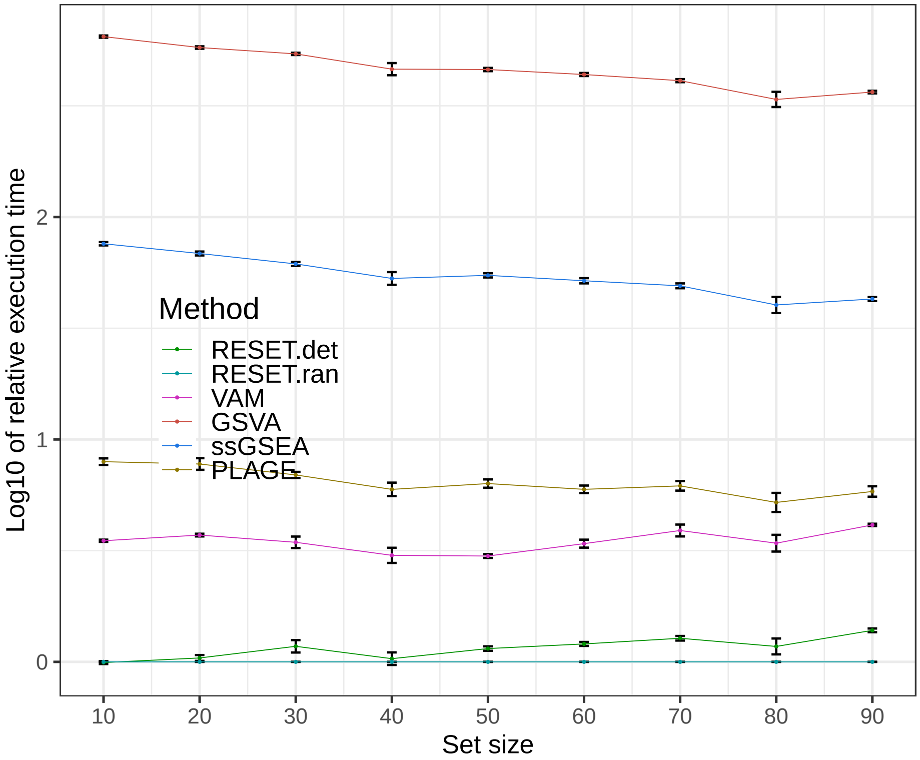

Figure D: Average execution time of RESET.det, VAM, GSVA, ssGSEA, and PLAGE relative to RESET.ran. Relative values are plotted on the $\log_{10}$ scale. Execution times were computed on data simulated according to the procedure outlined in Section 1.3 for the block design. Error bars represent the standard error of the mean.

Rank	Pathway	RESET score	Rank	Pathway	RESET score
1	BIOCARTA-MHC-PATHWAY	0.175	11	BIOCARTA-D4GDI-PATHWAY	0.061
2	BIOCARTA-UCALPAIN-PATHWAY	0.170	12	BIOCARTA-ASBCELL-PATHWAY	0.055
3	BIOCARTA-CTL-PATHWAY	0.140	13	BIOCARTA-THELPER-PATHWAY	0.055
4	BIOCARTA-TH1TH2-PATHWAY	0.137	14	BIOCARTA-FCER1-PATHWAY	0.054
5	BIOCARTA-IL17-PATHWAY	0.131	15	BIOCARTA-TCYTOTOXIC-PATHWAY	0.053
6	BIOCARTA-NPC-PATHWAY	0.126	16	BBIOCARTA-EICOSANOID-PATHWAY	0.051
7	BIOCARTA-MTA3-PATHWAY	0.098	17	BIOCARTA-DC-PATHWAY	0.049
8	BIOCARTA-TCRA-PATHWAY	0.092	18	BIOCARTA-CD40-PATHWAY	0.043
9	BIOCARTA-CCR5-PATHWAY	0.066	19	BIOCARTA-EIF-PATHWAY	0.043
10	BIOCARTA-CARDIACEGF-PATHWAY	0.062	20	BIOCARTA-ETC-PATHWAY	0.041

Table A: Top 20 BioCarta pathways according to overall RESET score for the PBMC data set with per-variable adjustment.

2.3 Human PBMC analysis

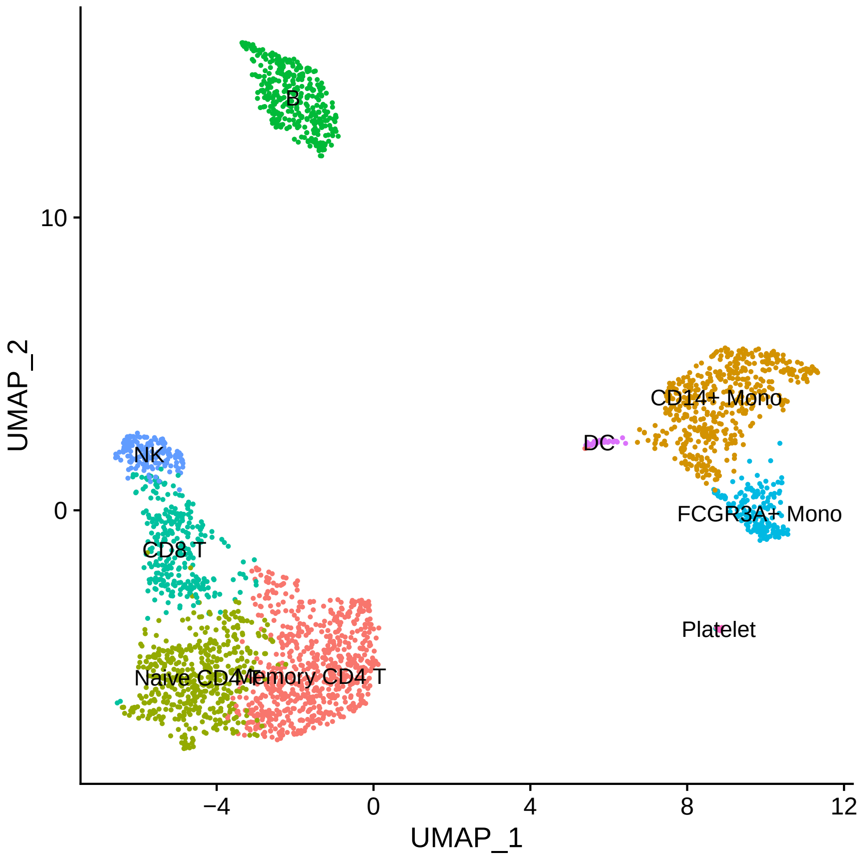

Figure E: Projection of PBMC scRNA-seq data onto the first two UMAP dimensions. Each point in the plot represents one cell. Cluster annotations reflect estimated cell type.

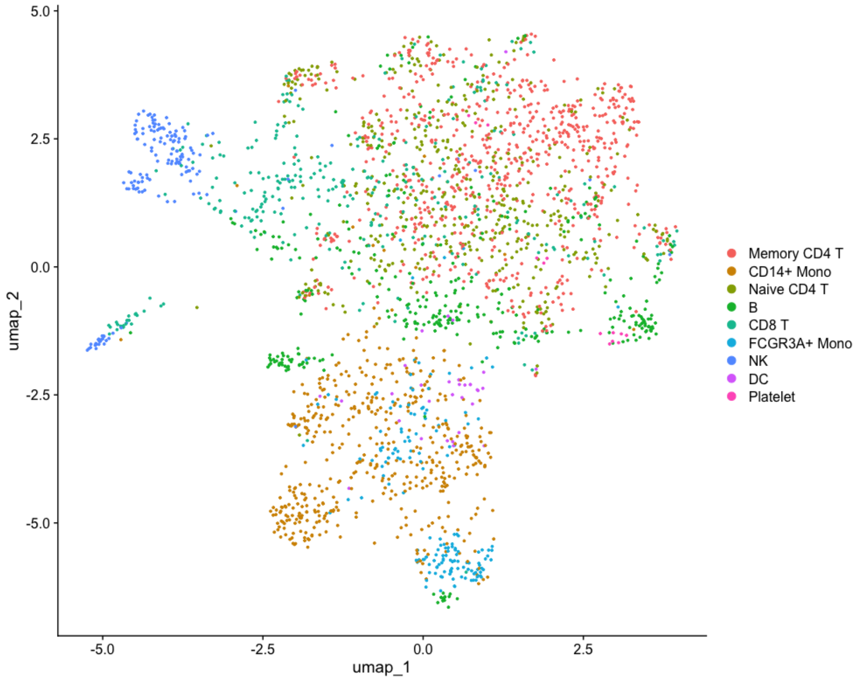

Figure F: Projection of RESET BioCarta scores for the PBMC scRNA-seq data onto the first two UMAP dimensions where UMAP was executed on the top 30 PCs of the RESET score matrix. Each point in the plot represents one cell.Annotations reflect estimated cell type according to the scRNA-seq clusters.

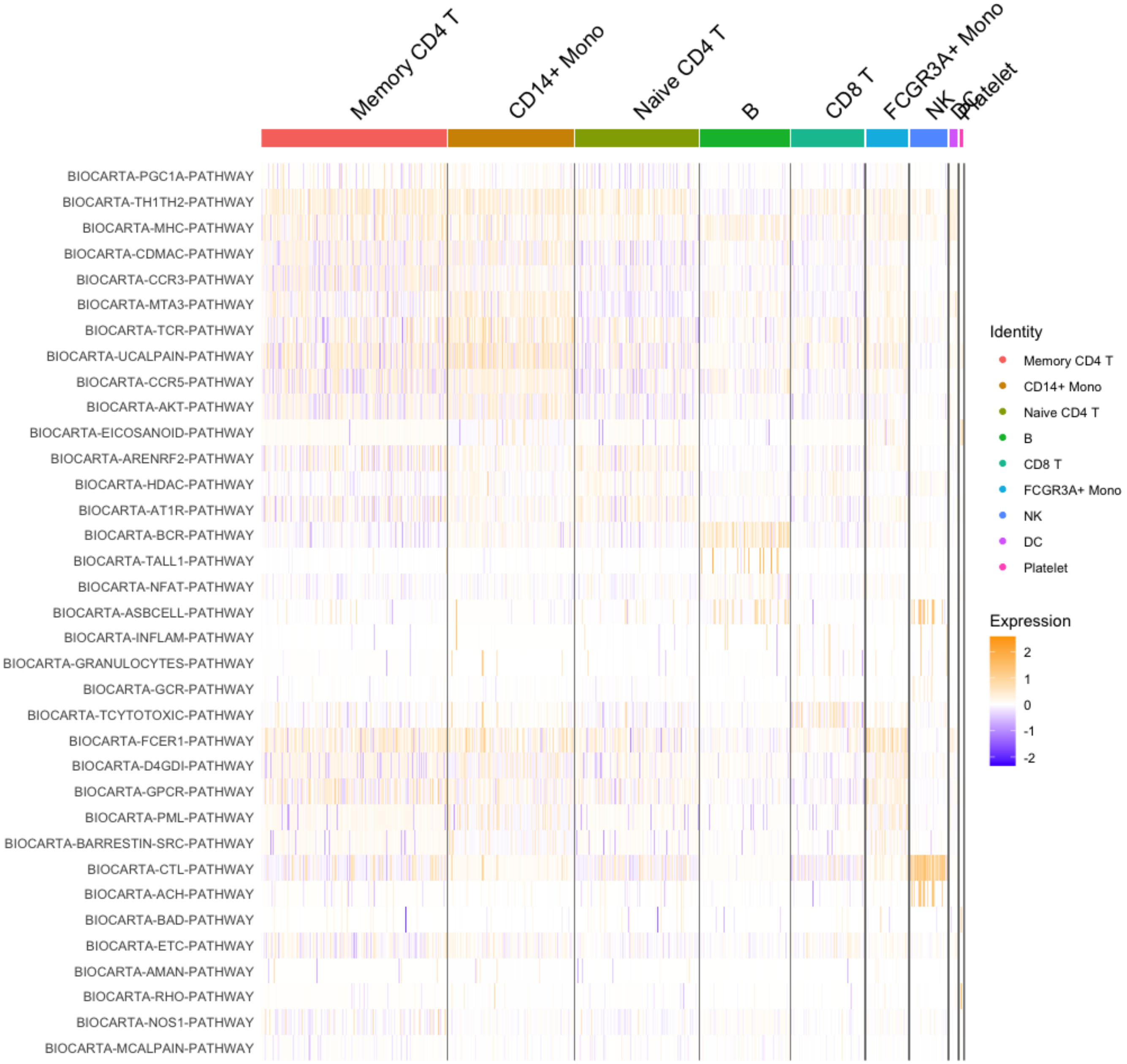

Figure G: Heatmap visualization of the RESET cell-specific scores for the top five BioCarta pathways most enriched in each cluster of the PBMC scRNA-seq data according to the log2 fold-change in the mean RESET score of cells in the cluster relative to cells not in the cluster. Note that gene sets only appear once in the heatmap even if they are among the top five sets for multiple clusters.

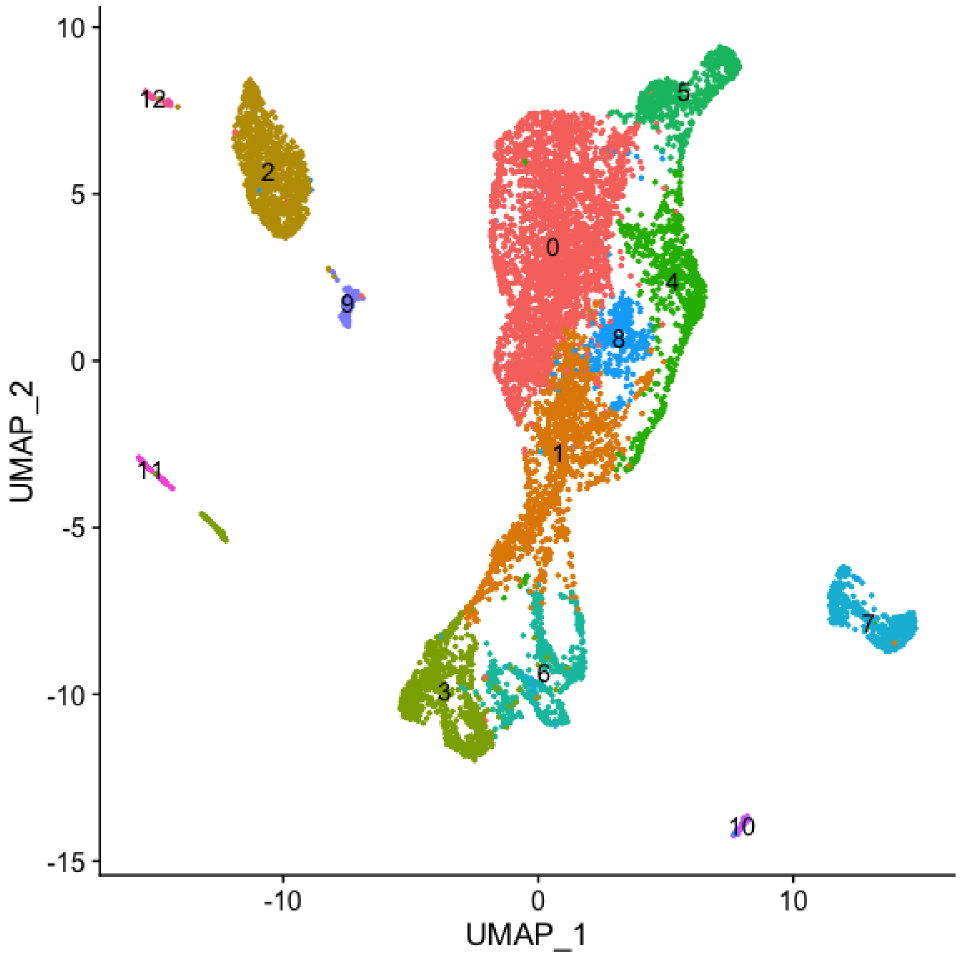

Figure H: Projection of mouse brain scRNA-seq data onto the first two UMAP dimensions. Each point in the plot represents one cell, which are colored and labeled accounting to the output from unsupervised clustering.

2.4 Mouse brain cell analysis

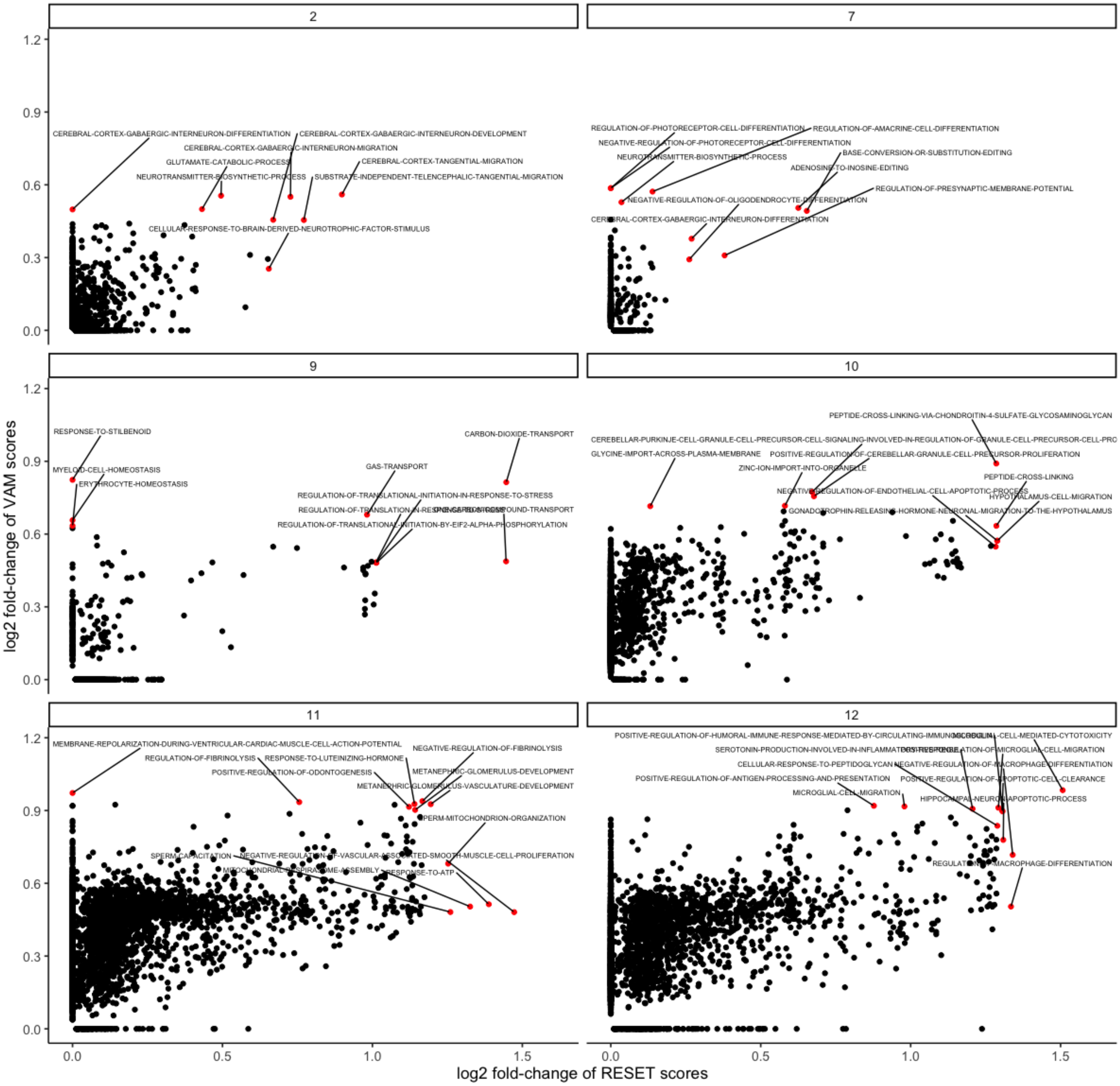

Figure I: Visualization of cluster-level Gene Ontology biological process term enrichment as computed using either VAM or RESET scores for the mouse brain scRNA-seq data.

Rank	Pathway	RESET score	Rank	Pathway	RESET score
1	BIOCARTA-TCRA-PATHWAY	0.155	11	BIOCARTA-RNA-PATHWAY	0.069
2	BIOCARTA-MHC-PATHWAY	0.137	12	BIOCARTA-INFR2-PATHWAY	0.066
3	BIOCARTA-INFLAM-PATHWAY	0.127	13	BIOCARTA-VIP-PATHWAY	0.066
4	BIOCARTA-TH1TH2-PATHWAY	0.091	14	BIOCARTA-BCR-PATHWAY	0.064
5	BIOCARTA-TOLL-PATHWAY	0.077	15	BIOCARTA-AKAPCENTROSOME-PATHWAY	0.059
6	BIOCARTA-D4GDI-PATHWAY	0.076	16	BBIOCARTA-CTL-PATHWAY	0.057
7	BIOCARTA-PGC1A-PATHWAY	0.073	17	BIOCARTA-TCR-PATHWAY	0.057
8	BIOCARTA-FCER1-PATHWAY	0.073	18	BIOCARTA-KERATINOCYTE-PATHWAY	0.056
9	BIOCARTA-SET-PATHWAY	0.071	19	BIOCARTA-TCYTOTOXIC-PATHWAY	0.054
10	BIOCARTA-NKCELLS-PATHWAY	0.070	20	BIOCARTA-THELPER-PATHWAY	0.054

Table B: Top 20 BioCarta pathways according to overall RESET score for the human cord blood data set using Seurat log-normalization.

Rank	Pathway	RESET score	Rank	Pathway	RESET score
1	BIOCARTA-TCRA-PATHWAY	0.239	11	BIOCARTA-THELPER-PATHWAY	0.084
2	BIOCARTA-MHC-PATHWAY	0.230	12	BIOCARTA-CTL-PATHWAY	0.081
3	BIOCARTA-INFLAM-PATHWAY	0.157	13	BIOCARTA-TNFR2-PATHWAY	0.077
4	BIOCARTA-SET-PATHWAY	0.131	14	BIOCARTA-PCAF-PATHWAY	0.068
5	BIOCARTA-RNA-PATHWAY	0.128	15	BIOCARTA-IL17-PATHWAY	0.068
6	BIOCARTA-D4GDI-PATHWAY	0.117	16	BBIOCARTA-DC-PATHWAY	0.066
7	BIOCARTA-PGC1A-PATHWAY	0.097	17	BIOCARTA-NKCELLS-PATHWAY	0.065
8	BIOCARTA-TH1TH2-PATHWAY	0.085	18	BIOCARTA-GATA3-PATHWAY	0.063
9	BIOCARTA-AKAPCENTROSOME-PATHWAY	0.084	19	BIOCARTA-TOLL-PATHWAY	0.060
10	BIOCARTA-TCYTOTOXIC-PATHWAY	0.084	20	BIOCARTA-AKAP95-PATHWAY	0.055

Table C: Top 20 BioCarta pathways according to overall RESET score for the human cord blood data set using Seurat log-normalization with per-variable adjustment.

2.5 Human cord blood cell analysis

Rank	Pathway	RESET score	Rank	Pathway	RESET score
1	BIOCARTA-MHC-PATHWAY	0.124	11	BIOCARTA-RNA-PATHWAY	0.070
2	BIOCARTA-CSK-PATHWAY	0.105	12	BIOCARTA-THELPER-PATHWAY	0.053
3	BIOCARTA-TCRA-PATHWAY	0.096	13	BIOCARTA-NO2IL12-PATHWAY	0.051
4	BIOCARTA-CD40-PATHWAY	0.095	14	BIOCARTA-CTCF-PATHWAY	0.049
5	BIOCARTA-BCR-PATHWAY	0.090	15	BIOCARTA-FCER1-PATHWAY	0.048
6	BIOCARTA-BLYMPHOCYTE-PATHWAY	0.089	16	BBIOCARTA-AHSP-PATHWAY	0.047
7	BIOCARTA-INFLAM-PATHWAY	0.080	17	BIOCARTA-CTLA4-PATHWAY	0.047
8	BIOCARTA-ASBCELL-PATHWAY	0.078	18	BIOCARTA-SET-PATHWAY	0.0047
9	BIOCARTA-ATM-PATHWAY	0.072	19	BIOCARTA-TFF-PATHWAY	0.046
10	BIOCARTA-CTL-PATHWAY	0.071	20	BIOCARTA-P38MAPK-PATHWAY	0.044

Table D: Top 20 BioCarta pathways according to overall RESET score for the human cord blood data set using SCTransform normalization.

Rank	Pathway	RESET score	Rank	Pathway	RESET score
1	BIOCARTA-MHC-PATHWAY	0.210	11	BIOCARTA-SET-PATHWAY	0.086
2	BIOCARTA-BLYMPHOCYTE-PATHWAY	0.147	12	BIOCARTA-IL17-PATHWAY	0.079
3	BIOCARTA-TCRA-PATHWAY	0.146	13	BIOCARTA-EPONFKB-PATHWAY	0.069
4	BIOCARTA-ASBCELL-PATHWAY	0.143	14	BIOCARTA-ATM-PATHWAY	0.069
5	BIOCARTA-CD40-PATHWAY	0.133	15	BIOCARTA-CARM1-PATHWAY	0.068
6	BIOCARTA-RNA-PATHWAY	0.129	16	BBIOCARTA-NO2IL12-PATHWAY	0.067
7	BIOCARTA-CSK-PATHWAY	0.106	17	BIOCARTA-AKAP95-PATHWAY	0.067
8	BIOCARTA-INFLAM-PATHWAY	0.104	18	BIOCARTA-AHSP-PATHWAY	0.066
9	BIOCARTA-CTL-PATHWAY	0.099	19	BIOCARTA-GATA3-PATHWAY	0.063
10	BIOCARTA-THELPER-PATHWAY	0.087	20	BIOCARTA-DREAM-PATHWAY	0.054

Table E: Top 20 BioCarta pathways according to overall RESET score for the human cord blood data set using SCTransform normalization with per-variable adjustment.

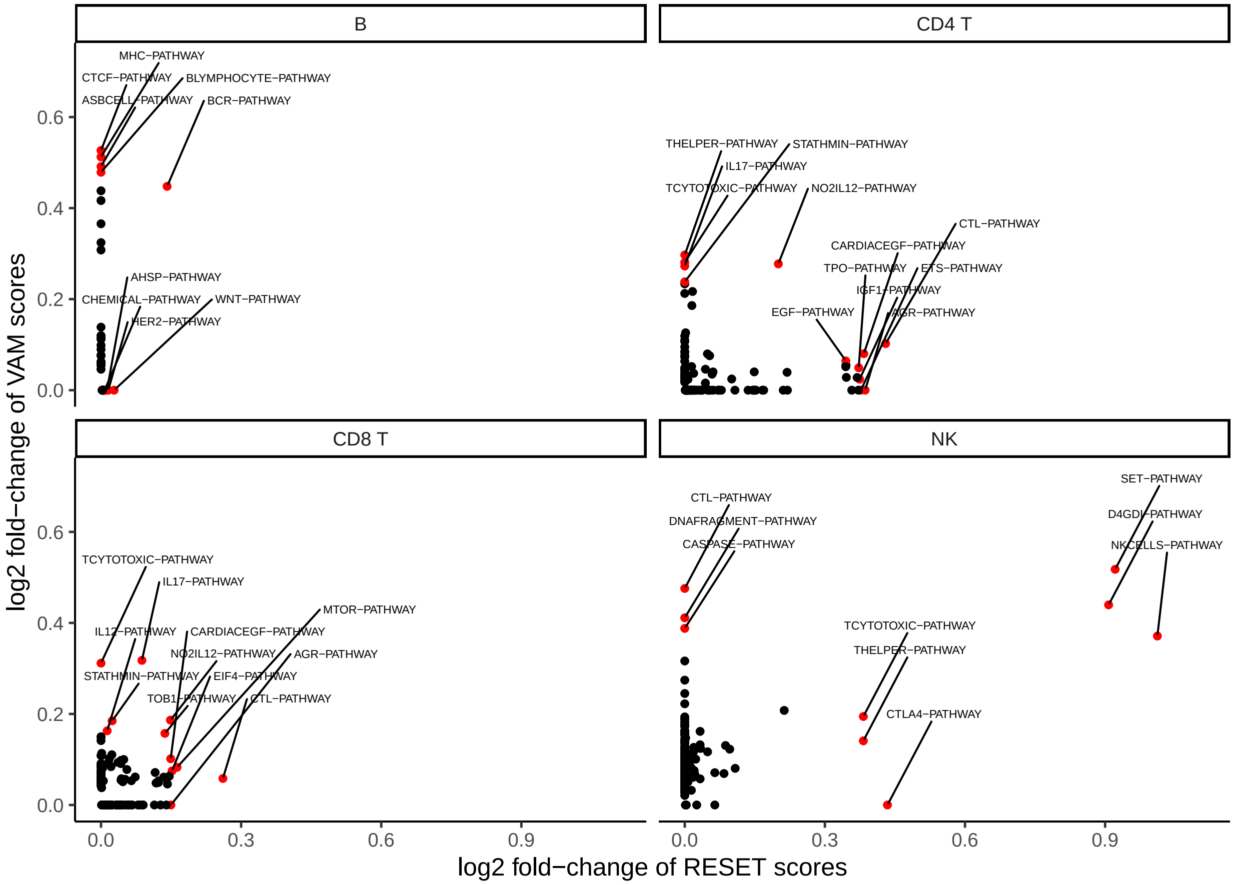

Figure J: Visualization of cell type BioCarta pathway enrichment for select immune cell types as computed using either VAM or RESET scores on the human cord blood scRNA-seq data using Seurat log-normalization.

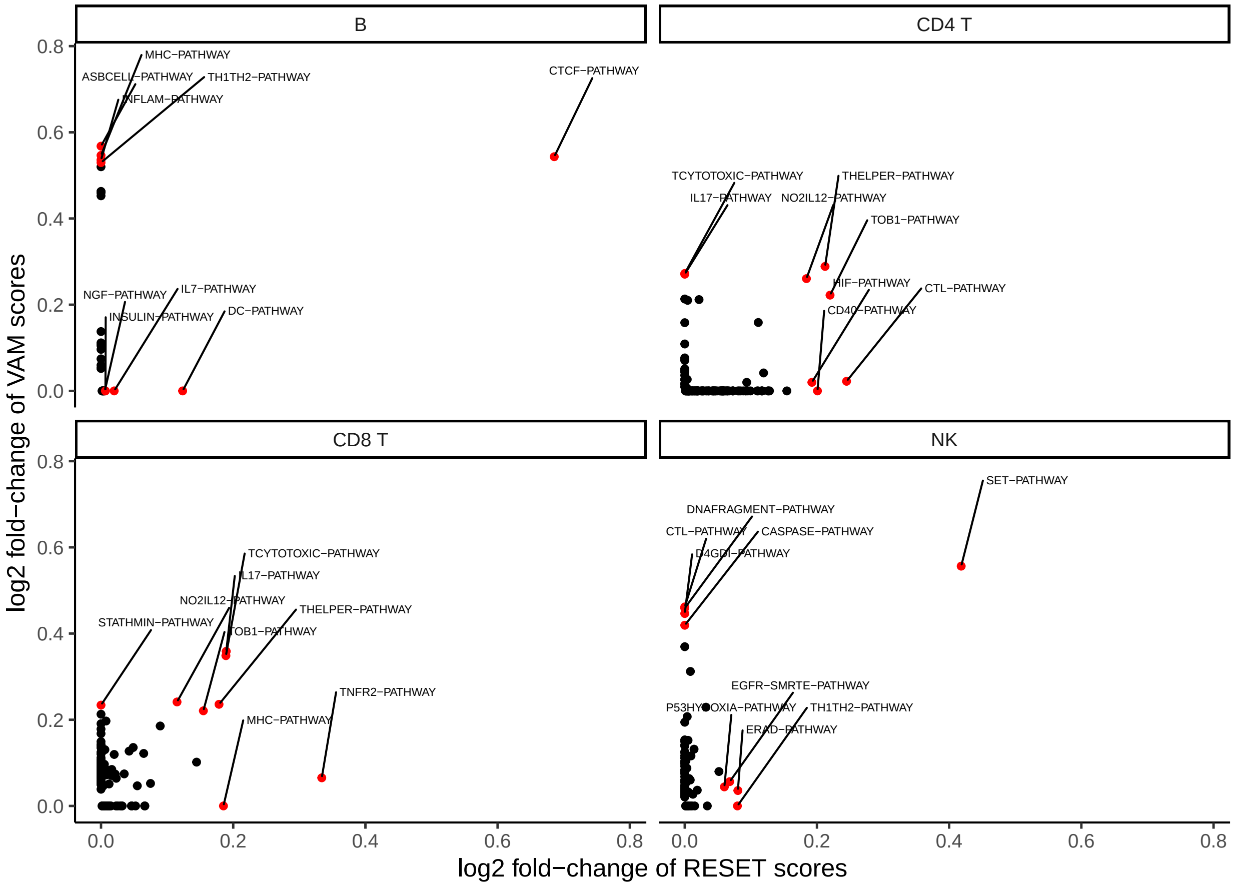

Figure K: Visualization of cell type BioCarta pathway enrichment for select immune cell types as computed using either VAM or RESET scores on the human cord blood scRNA-seq data using SCTransform normalization.

References

[1] Martinsson, P.-G., Tropp, J.A.: Randomized numerical linear algebra: Foundations and algorithms. Acta Numerica **29**, 403–572 (2020). doi:10.1017/S0962492920000021

[2] Erichson, N.B., Voronin, S., Brunton, S.L., Kutz, J.N.: Randomized matrix decompositions using r. Journal of Statistical Software **89**(11), 1–48 (2019). doi:10.18637/jss.v089.i11

[3] Stuart, T., Butler, A., Hoffman, P., Hafemeister, C., Papalexi, E., Mauck, W.M. 3rd, Hao, Y., Stoeckius, M., Smibert, P., Satija, R.: Comprehensive integration of single-cell data. Cell **177**(7), 1888–190221 (2019). doi:10.1016/j.cell.2019.05.031

[4] Hafemeister, C., Satija, R.: Normalization and variance stabilization of single-cell rna-seq data using regularized negative binomial regression. Genome Biol **20**(1), 296 (2019). doi:10.1186/s13059-019-1874-1

[5] Lause, J., Berens, P., Kobak, D.: Analytic pearson residuals for normalization of single-cell rna-seq umi data. Genome Biol **22**(1), 258 (2021). doi:10.1186/s13059-021-02451-7

[6] Seurat: Seurat Guided Clutering Tutorial. `https://satijalab.org/seurat/v3.1/pbmc3k_tutorial.html`. Accessed: 2020-02-10 (2020)

[7] 10x Genomics: 10k Brain Cells from an E18 Mouse (v3 chemistry). `https://support.10xgenomics.com/single-cell-gene-expression/datasets/3.0.0/neuron_10k_v3?` Accessed: 2020-02-10

[8] Carter, R.A., Bihannic, L., Rosencrance, C., Hadley, J.L., Tong, Y., Phoenix, T.N., Natarajan, S., Easton, J., Northcott, P.A., Gawad, C.: A single-cell transcriptional atlas of the developing murine cerebellum. Curr Biol **28**(18), 2910–29202 (2018). doi:10.1016/j.cub.2018.07.062

[9] McInnes, L., Healy, J., Melville, J.: UMAP: Uniform Manifold Approximation and Projection for Dimension Reduction (2018). 1802.03426

[10] Waltman, L., van Eck, N.J.: A smart local moving algorithm for large-scale modularity-based community detection. The European Physical Journal B **86**(11) (2013). doi:10.1140/epjb/e2013-40829-0

[11] Liberzon, A., Subramanian, A., Pinchback, R., Thorvaldsdóttir, H., Tamayo, P., Mesirov, J.P.: Molecular signatures database (msigdb) 3.0. Bioinformatics **27**(12), 1739–40 (2011). doi:10.1093/bioinformatics/btr260

[12] Gene Ontology Consortium: The gene ontology in 2010: extensions and refinements. Nucleic Acids Res **38**(Database issue), 331–5 (2010). doi:10.1093/nar/gkp1018
