# Supplementary material for: Reconstruction Set Test (RESET): A computationally efficient method for single sample gene set testing based on randomized reduced rank reconstruction error
Source: PLoS Comput Biol. 2024 Apr 29;20(4):e1012084. doi: 10.1371/journal.pcbi.1012084 (PMC11081506; doi:10.1371/journal.pcbi.1012084)
Supplement: Attachment — Submitted filename: RESET_response_to_comments.pdf [file pcbi.1012084.s002.pdf]

Response to reviewer comments for "Reconstruction Set Test (RESET): a computationally efficient method for single sample gene set testing based on randomized reduced rank reconstruction error"

H. Robert Frost

I appreciate the thoughtful and helpful feedback from the reviewers. Responses to each of the reviewer's comments are outlined in the sections below (reviewer comments are in bold and my response follows in normal font). In addition to the revised manuscript (now in the PLOS LaTeX template) and SI, the resubmission also includes files showing the differences between these revised versions and the original versions, as computed via latexdiff (note: to avoid capturing PLOS template formatting changes, the diff was computed prior to migration to the PLOS template; also, some minor manual editing of the diff tex file was necessary, primarily around citations, to avoid compilation errors).

I believe that addressing the reviewer's comments has substantially improved the revised manuscript by:

- Expanding the background section to more clearly articulate the motivation for the RESET method and relevant prior work.
- Adding a workflow figure for the RESET method and clarifying other aspects of the technique.
- Expanding the simulation study to include results from a PCA-based technique (PLAGE) and results for a more complex model of gene set correlation and mean abundance (new "complex" simulation model).
- Clarifying the interpretation of the analysis results.

Although not related to the reviewer's comments, the RESET R package has also been updated on CRAN (now v1.0.0) to support integration with Seurat v5.0.

1 Reviewer 1 Comments

In this study, the authors developed a randomized numerical linear algebra (RNLA) based computational framework, RESET, for the unsupervised single sample gene set testing. By leveraging the RNLA, the authors aimed to provide a computationally efficient tool. The authors evaluated the performance of REST on both simulated and real single-cell datasets. Although the proposed computational framework is interesting, I have major concerns as stated below:

1.1 Reviewer 1 Major Comments

1. **The main innovation of REST is the use of RNLA for computationally efficient dimensionality reduction. However, this improvement in efficiency over PCA may be marginal. Since the computational cost of PCA is dominated by the small dimension of X (e.g., min (n,p)). Also, n can become large, and the dimensionality of p tends to be ∼2000 (e.g. for highly variable genes). It seems to me that deterministic PCA will work for most of the task, and that RNLA is not necessary.**

The reviewer raises an excellent point regarding the expected performance benefit of RNLA vs. deterministic matix decomposition algorithms such as truncated PCA in many practical scenarios. There are several points to consider on this topic:

- While computational performance is an important benefit of the RESET method, the main innovation is the use of reconstruction error to support both population-level and single sample gene set testing; these benefits hold even if a purely deterministic algorithm is used, e.g., Algorithm 2 in the paper which does not incorporate RNLA. Results for this non-randomized version of RESET are represented by the RESET.det method in the results in the paper.
- Even when RNLA is not used in the RESET method (i.e., the RESET.det results in the manuscript), RESET still has a significant performance benefit relative to comparable techniques as shown in Table 1 and SI Figure S4.
- The RESET method does support the use randomized PCA vs. trucated PCA for overall dimensionality reduction and the reviewer is correct that the performance benefit of RNLA for this step is reduced when applied to only the most variable genes. However, overall dimensionality reduction is only a small part of the total execution time for RESET, which is dominated by the dimensionality reduction of the sub-matrices corresponding to each gene set. It is also worth noting that the commonly performed restriction to highly variable genes is partly motivated by the performance limitations of deterministic PCA algorithms; using RNLA for this step avoids the need to filter genes before overall dimensionality reduction.

2. **The working principles of REST and its ancestor VAM on single sample gene set testing are not explained. The writing style, especially the methods part, is more like a manual. I faced some challenges in understanding the manuscript.**

   The reviewer raises a good point, which was also identified by Reviewer 2 in their minor comment #1. To address this issue, I have updated the background section to provide more detail on gene set testing methods and the motivation for single sample techniques such as VAM and RESET. The new workflow figure (see response to minor comment #1 below) should also help improve the readability of the methods section.

3. **The parameters used in the performance evaluation need to be explained. For example, "classification" of what? Why can "classification" be used for gene set testing? what do "mean inflation", "correlation", "rate" mean?**

   The reviewer raises an excellent point. In the original submission, the detailed description of these simulation parameters and the associated simulation framework was contained in SI Section 1.3. To help readers better interpret the simulation results, condensed descriptions of these parameters are now included in Section 3.1 of the main manuscript. Regarding the interpretation of "classification", this is quantified for the simulation results as the area under the receiver operating characteristic curve (AUC) and captures the ability of each evaluated method to generate high scores for the informative gene set in informative cells, i.e, cells whose simulated counts have higher mean expression and/or non-zero correlation between genes in the informative gene set.

   If the reviewer feels that the material in SI Section 1.3 is needed in the main manuscript, I am open to making that change.

1.2 Reviewer 1 Minor Comments

1. **I suggest that a diagram of the RESET workflow be provided. Some details on the RNLA used would also be helpful.**

The reviewer makes a great suggestion and a workflow visualization of the RESET method (as defined by Algorithm 3) is now included as Figure 1 in the manuscript. This workflow figure has been included inline below.

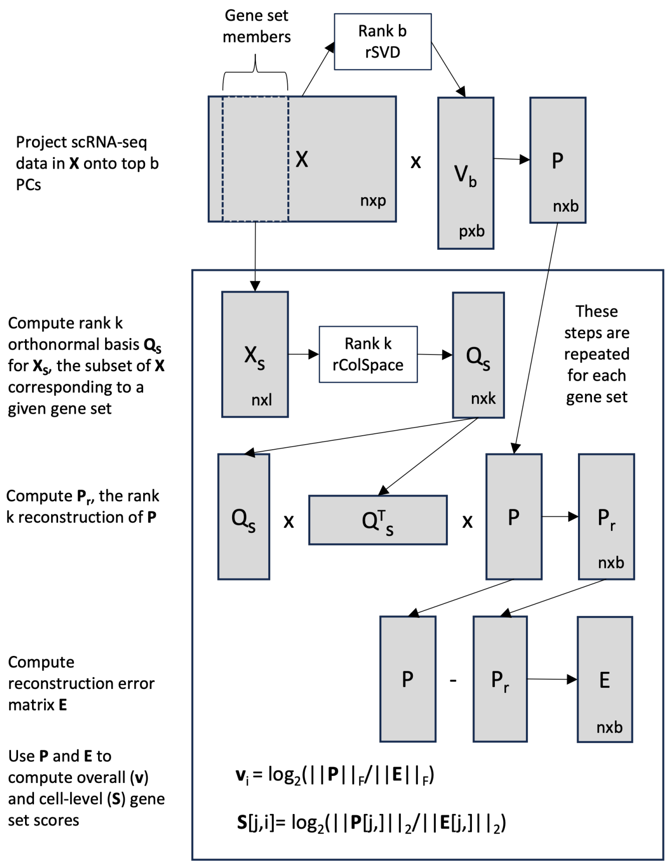

Figure 1: Workflow representation of the randomized version of RESET as defined by Algorithm 3.

The reviewer also raises an excellent point regarding RNLA details. The current version of the manuscript has most of the detailed RNLA material in Section 1.1 of the SI. I have updated the main manuscript to reinforce that readers unfamiliar with RNLA should review SI Section 1.1 and, for more information, read the paper by Erichson et al [1] associated with the *rsvd* R package and the more detailed survey by Martinsson et al. [2]. If the reviewer feels that material in SI Section 1.1 would be more helpful in the main manuscript, I am open to moving that discussion and SI Algorithms S1 and S2 into the main body of the paper.

2. **How does RESET deal with the batch effect when samples are collected and sequenced in different batches?**

   The reviewer raises a good question. In short, the RESET method itself does not directly address batch effects and assumes that appropriate normalization/batch correction has been performed earlier in the analysis pipeline. I have added a note Section 2.1 of the revised manuscript to clarify this issue.

2 Reviewer 2 Comments

The paper proposed a new method called Reconstruction Set Test (RESET) for single sample gene set testing. RESET quantifies the importance of gene sets based on their ability to reconstruct values for all measured genes. It uses randomized reduced rank reconstruction

algorithm to detect patterns of differential abundance and differential correlation for both self-contained and competitive scenarios. The method generates both overall and sample-level scores for evaluated gene sets. However, there are some issues, which must be solved before it is considered for publication.

2.1 Reviewer 2 Major Comments

1. **Why the RNLA, more than PCA and Z-score[1], is effective in detecting patterns of differential abundance and differential correlation in independent and competing scenes(In section 2.1).**

   The reviewer asks a great question. One of the key factors underlying the relative benefit of RESET vs. techniques like VAM, the z-scoring method or the PCA-based PLAGE technique (which we have now included in the results, see response to major comment #2 below) is that VAM, the z-scoring method and PLAGE are all self-contained methods. In other words, these methods only consider expression values for genes in the tested set and ignore the values for other measured genes. This has the consequence that these techniques can identify self-contained patterns of differential abundance but struggle to detect competitive cases (e.g., the results for the pure competitive simulation design shown in the original Figure 4, which is now Figure 6 in the revised manuscript). Additionally, techniques like VAM and z-scoring are designed to detect differences in mean expression so perform poorly when there is differential correlation without significant differential expression (e.g., see the results in the first two panels of Figure 1 when mean inflation is close to 1 and when differential correlation is high). We have updated the text in the revised results section to help clarify these distinctions.

2. **More experiments should be offered to demonstrate the validity and applicability of the proposed method. For example, PLAGE and PAGODA are added to the comparison methods and explain why the RESET can detect competitive scenarios where the measured values of set genes differ from non-set genes in the same sample.**

   The reviewer makes an excellent suggestion regarding additional experiments and comparison methods. To address this, I have expanded the simulation study to:

   - Include results from a self-contained PCA-based technique that is sensitive to patterns of differential correlation. I have specifically added results for the PLAGE [3] method to all of the simulation study results. Both PLAGE and PAGODA [4] are PCA-based techniques that generate gene set scores using the projection of each sample/cell onto the first PC of the sub-matrix corresponding to the evaluated gene set. I choose to use PLAGE as the example PCA-based technique rather than PAGODA given the relative simplicity of the PLAGE technique and stable implementation in the GSVA R package (PAGODA requires a custom normalization process and first assesses overall PC significance using a Tracey-Widom test on the associated eigenvalue). While techniques like PLAGE and PAGODA are sensitive to patterns of differential correlation, they only use the first PC of the gene set expression data so work poorly when the relevant biological signal is spread across multiple PCs (or represented by a single PC that is not associated with the largest eigenvalue, which is the case for the new complex simulation design). By contrast, RESET takes into account the rank k representation of the gene set expression data.
   - Include a more complex simulation model that splits the evaluated gene set into two correlated blocks with distinct patterns of mean expression. While the experimental expression data for many gene sets may be effectively modeled using just a single PC, the biologically relevant signal for a gene set can often be spread across 2 or more PCs. This new model captures the not uncommon scenario of two non-random PCs. The results for this new model, which is referred to as the "complex" model, are included as the new Figure 3 in the revised manuscript with simulation details in SI Section 1.3. The relevant biological signal for this model is captured

by PC 2 rather than PC 1, which significantly limits the utility of techniques like PLAGE and PAGODA that leverage only the first PC.

The reviewer also asks a great question regarding why RESET can detect competitive scenarios where the measured values of set genes differ from non-set genes in the same sample. The RESET method uses a reduced rank representation of a single gene set to reconstruct a reduced rank representation of the entire expression matrix. In a competitive scenario where set genes have greater correlation/mean expression than non-set genes, this reconstruction process will be more successful than for cases where set members have a very similar distribution as non-set members. As an extreme example, one can imagine a gene expression matrix that has just a single non-random PC that is associated with a single gene set (i.e., the population covariance matrix has a single block associated with the set genes); in that case, a reduced rank representation of the gene set will be very effective in reconstructing a reduced rank representation of the entire expression matrix. I have expanded the last section of the Background (now titled "Gene set testing based on reconstruction error") to help clarify this issue.

3. **The simulation results should be explained in detail, such as why the higher inter-gene correlation indicates the better the RESET results**

   Thank you for raising this point and I have expanded the discussion of the simulation results in the revised manuscript. I have specifically added text near the beginning of the simulation results section that provides more detail on the two main performance trends observed on the simulation model: 1) performance relative to mean inflation of informative genes in informative cells, and 2) performance relative to the level of correlation between informative genes in informative cells.

   Regarding the reviewer's specific question about why RESET performance improves with the level of inter-gene correlation, this is due to the fact that as a subset of variables becomes more correlated relative to all variables in the matrix, those variables will become more associated with a reduced rank representation of the matrix. In the extreme case where there is just a single informative PC for a matrix (i.e., the non-random signal in the matrix is due to a single correlated block in the covariance matrix), the set of variables in the correlated block can effectively reconstruct the entire matrix. The reason why most other single sample methods do not see a similar trend is due to both the self-contained nature of many tests and, for all evaluated techniques except for PLAGE, a focus on differential abundance vs. differential correlation.

2.2 Reviewer 2 Minor Comments

1. **The Introduction section does not provide sufficient background information or context to help readers understand the significance of your research. We suggest that you consider adding more detail, such as what are the competitive and self-contained features.**

   The reviewer raises a great point that was also identified by Reviewer 1 in their major comment #2. To address these comments, I have expanded the introduction to provide more detail on gene set testing methods and the motivation for single sample techniques such as VAM and RESET.

References

[1] N. Benjamin Erichson, Sergey Voronin, Steven L. Brunton, and J. Nathan Kutz. Randomized matrix decompositions using r. *Journal of Statistical Software*, 89(11):1–48, 2019.

[2] Per-Gunnar Martinsson and Joel A. Tropp. Randomized numerical linear algebra: Foundations and algorithms. *Acta Numerica*, 29:403–572, 2020.

[3] John Tomfohr, Jun Lu, and Thomas B Kepler. Pathway level analysis of gene expression using singular value decomposition. *BMC Bioinformatics*, 6:225, 2005.

[4] Jean Fan, Neeraj Salathia, Rui Liu, Gwendolyn E Kaeser, Yun C Yung, Joseph L Herman, Fiona Kaper, Jian-Bing Fan, Kun Zhang, Jerold Chun, and Peter V Kharchenko. Characterizing transcriptional heterogeneity through pathway and gene set overdispersion analysis. *Nat Methods*, 13(3):241–4, Mar 2016.
