# Supplementary material for: Reconstruction Set Test (RESET): A computationally efficient method for single sample gene set testing based on randomized reduced rank reconstruction error
Source: PLoS Comput Biol. 2024 Apr 29;20(4):e1012084. doi: 10.1371/journal.pcbi.1012084 (PMC11081506; doi:10.1371/journal.pcbi.1012084)
Supplement: Attachment — Submitted filename: RESET_response_to_comments.pdf [file pcbi.1012084.s003.pdf]

Response to reviewer comments on R1 version of "Reconstruction Set Test (RESET): a computationally efficient method for single sample gene set testing based on randomized reduced rank reconstruction error"

H. Robert Frost

I appreciate the thoughtful feedback from the reviewers on the revised manuscript. Responses to each of the reviewer's comments are outlined in the sections below (reviewer comments are in bold and my response follows in normal font). In addition to the revised manuscript and SI, the resubmission also includes files showing the differences between first and second revision as computed via latexdiff (note: some minor manual editing of the diff tex file as necessary, primarily around citations, to avoid compilation errors).

1 Reviewer 1 Comments

My concerns have been addressed.

2 Reviewer 2 Comments

The authors have revised the paper carefully according to the comments. I think the paper can be accepted in the current version.

3 Reviewer 3 Comments

The author(s) developed a new gene set testing method RESET, which quantifies gene set importance for transcriptomics datasets with multiple samples/conditions. The implementation of RESET was introduced clearly by first setting the basic RESET method of constructing the full expression matrix with a subset of genes in a gene set, and then adding in the rank reduction through PCA component, and finally adding randomized numerical linear algebra for increased computational efficiency. The revised manuscript provides sufficient motivation for RESET in the introduction, and improved explanation of the improvements of RESET over existing methods. The method will be useful for interpretation of transcriptomics datasets. I have only one major comment and a few minor comments/questions.

3.1 Reviewer 3 Major Comments

1. **It would help to clarify more up front that for single cell datasets the scores are calculated on each cell. The term sample could mean the individual replicates, and indicate that the RESET scores are calculated on pseudobulk samples.**

   Thank you for the suggestion. I have added clarifying text to the "Single sample gene set testing challenges" section in the Introduction and to the start of the methods section to clarify that the n

samples captured in the input **X** matrix can represent either bulk tissue samples or individual cells depending on the nature of the dataset.

2. **Since RESET calculates cell level scores, a natural comparison would be AUCell from the SCENIC pipeline. Does RESET scores correlate with AUCell scores well? Are RESET scores more informative than AUCell scores?**

   The reviewer asks a great question and I have updated the "Single sample gene set testing" section in the Introduction to include a description of the AUCell method and how it relates to other single sample techniques. Similar to GSVA and ssGSEA, the AUCell [1] method generates sample/cell-level gene set scores based on gene ranks within each sample/cell. Specifically, AUCell uses a simple "area under the curve" (AUC) metric to quantify the enrichment of highly expressed genes within each evaluated set for a given sample/cell. This metric is effectively the proportion of top ranked genes in the sample/cell that are contained in the evaluated gene set (as stated in the AUCell documentation: "In a simplified way, the AUC value represents the fraction of genes, within the top X genes in the ranking, that are included in the signature"). In contrast to GSVA and ssGSEA, AUCell does not specifically account for set size or the distribution of gene expression values across all cells/samples in the data set. In terms of the relative performance, AUCell is similar to GSVA and ssGSEA on the classification performance metrics but with better computational performance given the fact that each cell is evaluated independently using an easy to compute metric. Given the similarities between AUCell, GSVA and ssGSEA, the fact that GSVA and ssGSEA are more sophisticated methods with a much wider adoption across both bulk and single cell data analysis problems, and the fact that the result plots are a bit crowded already given the number of evaluated method (3 versions of RESET, VAM, GSVA, ssGSEA and PLAGE), I felt it was sufficient to include results for just GSVA and ssGSEA as examples of competitive rank-based single sample techniques. However, if the reviewer feels it is important to also include AUCell in the simulation results, I am open to making those changes.

3. **Is there intuition on how deeply sequenced each cell needs to be (or the sparsity level of the dataset) for RESET to perform effectively?**

   That is an excellent question. While there is not a specific minimum required sequencing depth (or maximum overall sparsity), the performance of RESET (and other single sample gene set testing methods) will generally improve as sequencing depth increases (and sparsity decreases) though actual performance at a given overall sparsity level can vary considerably depending on sparsity levels of the genes in the tested set. This is approximately captured in the simulation results by the mean and rate parameters (see SI Section 1.3 for a detailed description of how these are used for simulating counts). As expected, performance for RESET generally improves as the mean increases. In general, I tried to structure the simulation studies to mirror the sequencing depth/sparsity found in real scRNA-seq datasets generated using platforms like 10x Chromium.

4. **For the real single cell datasets, if the cell by gene set scores matrix is projected and visualized, or clustered, do cells of the same type still cluster together?**

   The reviewer asks an excellent question. To explore this, I computed the projection of the RESET scores for the BioCarta pathway collection and the PBMC scRNA-seq data and have included this as Figure S6 in S1 Text (the standard UMAP projection is Figure S5). I have included this figure below for easy reference. The PBMC dataset used in the Seurat Guided Clustering tutorial and has the benefit in this context of clustering well according to cell type with a clear separation in the first two UMAP dimensions (see Figure S5). When the UMAP projection is computed using RESET scores (specifically on the top 30 PCs of the RESET score matrix), the clean clustering by cell type seen in the scRNA-seq UMAP projection is lost though there is still a visible segregation by cell type with some types, e.g., NK cells, retaining a clear separation from the other cells. The blurring of cell type identity is not surprising in this case given the more limited set of genes included in the analyzed

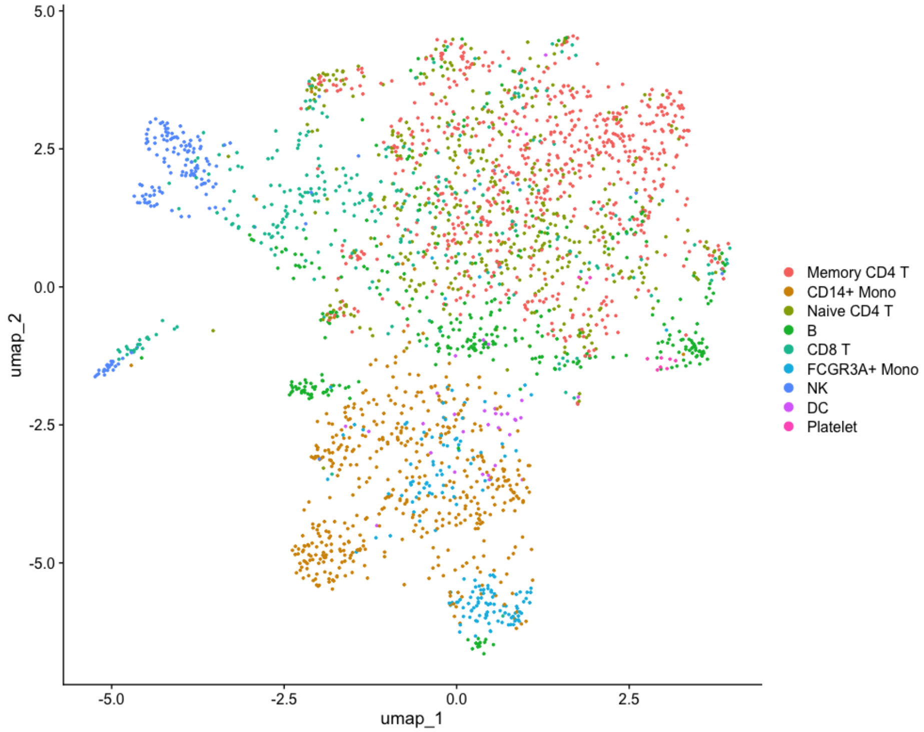

Figure 1: Projection of RESET BioCarta scores for the PBMC scRNA-seq data onto the first two UMAP dimensions where UMAP was executed on the top 30 PCs of the RESET score matrix. Each point in the plot represents one cell. Annotations reflect estimated cell type according to the scRNA-seq clusters.

BioCarta pathways and the fact that RESET scores capture reconstruction error rather than mean abundance.

5. **I think the possible ways to utilize cell level RESET scores for interpretation of single cell datasets should be mentioned in the methods section.**

   The reviewer makes a great suggestion and I have moved the "Usage considerations" section from the Results to the Methods.

3.2 Reviewer 3 Minor Comments

1. **Are all genes in X required to be in at least one gene set?**

   The reviewer asks a good question. No, all of the p genes in matrix $\mathbf{X}$ do not need to be in at least one gene set. I have updated the methods text where the gene set annotation matrix $\mathbf{A}$ is introduced to clarify this point.

2. **A few terms could be better introduced in the text:**

   (a) **When the concept of 'Self-contained vs competitive' was first introduced in section1.1, the definition was a more mathematical one, while in section1.2 the 'Self-contained methods' is described in more layman terms 'generate scores using only the data for genes in the set'. I think it'd help to make a clear connection between these descriptions or consolidate.**

   Thank you for the suggestion. I have updated the text in Section 1.1 ("Gene set testing") to also include a more intuitive description of self-contained and competitive techniques.

(b) **The term 'classification performance' is still somewhat confusing. I think more clearly defining the classification task, if there is one, would help.**

Thank you for the suggestion. I have updated the text where the term "classification performance" is first mentioned to clarify the interpretation, i.e., the ability of a given gene set testing method to assign high gene set scores to cells/samples with inflated expression of set genes.

(c) **The term 'differential correlation' was mentioned early on but not defined until the results section (where correlation was explained but still not clearly defined).**

Thank you for identifying this point. I have updated the text where "differential correlation" is first mentioned to clarify the interpretation.

3. **Is novel gene set discovery a possibility within this framework?**

   Great question. While this is not something I have explored, it is an intriguing idea and two possible approaches may be feasible:

   (a) **Gene set optimization**: Start with an existing gene set and remove members to maximize the per-variable overall RESET score.

   (b) **De novo gene set discovery**: Generate initial gene sets via some unsupervised analysis of the sample covariance matrix (e.g., gene clustering using correlation distance, or sparse PCA) then refine these initial sets via gene remove (or potentially addition, though that is more computationally challenging) to maximize the per-variable overal RESET score.

   I have updated the Conclusion section to mention these as potential future directions.

References

[1] Sara Aibar, Carmen Bravo González-Blas, Thomas Moerman, Vân Anh Huynh-Thu, Hana Imrichova, Gert Hulselmans, Florian Rambow, Jean-Christophe Marine, Pierre Geurts, Jan Aerts, Joost van den Oord, Zeynep Kalender Atak, Jasper Wouters, and Stein Aerts. Scenic: single-cell regulatory network inference and clustering. *Nat Methods*, 14(11):1083–1086, Nov 2017.
